# The health and economic burden of bloodstream infections caused by antimicrobial-susceptible and non-susceptible Enterobacteriaceae and *Staphylococcus aureus* in European hospitals, 2010 and 2011: a multicentre retrospective cohort study

**DOI:** 10.2807/1560-7917.ES.2016.21.33.30319

**Published:** 2016-08-18

**Authors:** Andrew J Stewardson, Arthur Allignol, Jan Beyersmann, Nicholas Graves, Martin Schumacher, Rodolphe Meyer, Evelina Tacconelli, Giulia De Angelis, Claudio Farina, Fabio Pezzoli, Xavier Bertrand, Houssein Gbaguidi-Haore, Jonathan Edgeworth, Olga Tosas, Jose A Martinez, M Pilar Ayala-Blanco, Angelo Pan, Alessia Zoncada, Charis A Marwick, Dilip Nathwani, Harald Seifert, Nina Hos, Stefan Hagel, Mathias Pletz, Stephan Harbarth, the TIMBER Study Group

**Affiliations:** 1 Infection Control Program, University of Geneva Hospitals and Faculty of Medicine, Geneva, Switzerland; 2 Department of Medicine, University of Melbourne, Melbourne, Australia; 3 Institute of Statistics, Ulm University, Ulm, Germany; 4 Institute of Medical Biometry and Medical Informatics, University Medical Center Freiburg, Freiburg, Germany; 5 Institute of Health and Biomedical Innovation, Queensland University of Technology, Brisbane, Australia; 6 Information Technology, University of Geneva Hospitals and Faculty of Medicine, Geneva, Switzerland; 7 Division of Infectious Diseases, Agostino Gemelli Hospital, Rome, Italy; 8 Division of Infectious Diseases, DZIF TTU-HAARBI, University Hospital Tübingen, Tübingen, Germany; 9 Papa Giovanni XXIII Hospital, Bergamo, Italy; 10 Centre hospitalier régional et universitaire (CHRU) Besançon, Besançon, France; 11 Department of Infectious Diseases, Kings College London, London, United Kingdom; 12 Hospital Clinic de Barcelona, Barcelona, Spain; 13 Istituti Ospitalieri di Cremona, Cremona, Italy; 14 Department of Infection and Immunodeficiency, Ninewells Hospital and Medical School, Dundee, United Kingdom; 15 Uniklinik Köln, Cologne, Germany; 16 German Centre for Infection Research (DZIF), Braunschweig, Germany; 17 Center for Infectious Diseases and Infection Control, University Hospital Jena, Jena, Germany; 18 The members of the group are listed at the end of the article

**Keywords:** Antimicrobial resistance, bacterial infections, bloodstream infection, *Staphylococcus aureus*, Meticillin-resistant *Staphylococcus aureus* (MRSA) in humans, *Escherichia coli*, multidrug resistance

## Abstract

We performed a multicentre retrospective cohort study including 606,649 acute inpatient episodes at 10 European hospitals in 2010 and 2011 to estimate the impact of antimicrobial resistance on hospital mortality, excess length of stay (LOS) and cost. Bloodstream infections (BSI) caused by third-generation cephalosporin-resistant Enterobacteriaceae (3GCRE), meticillin-susceptible (MSSA) and -resistant *Staphylococcus aureus* (MRSA) increased the daily risk of hospital death (adjusted hazard ratio (HR) = 1.80; 95% confidence interval (CI): 1.34–2.42, HR = 1.81; 95% CI: 1.49–2.20 and HR = 2.42; 95% CI: 1.66–3.51, respectively) and prolonged LOS (9.3 days; 95% CI: 9.2–9.4, 11.5 days; 95% CI: 11.5–11.6 and 13.3 days; 95% CI: 13.2–13.4, respectively). BSI with third-generation cephalosporin-susceptible Enterobacteriaceae (3GCSE) significantly increased LOS (5.9 days; 95% CI: 5.8–5.9) but not hazard of death (1.16; 95% CI: 0.98–1.36). 3GCRE significantly increased the hazard of death (1.63; 95% CI: 1.13–2.35), excess LOS (4.9 days; 95% CI: 1.1–8.7) and cost compared with susceptible strains, whereas meticillin resistance did not. The annual cost of 3GCRE BSI was higher than of MRSA BSI. While BSI with *S. aureus* had greater impact on mortality, excess LOS and cost than Enterobacteriaceae per infection, the impact of antimicrobial resistance was greater for Enterobacteriaceae.

## Introduction

Antimicrobial resistance (AMR) represents a significant global threat [1,2]. Response to this threat requires coordinated international interventions likely to involve commitment of substantial resources [3]. It is useful to obtain accurate estimates of the health and economic burden of AMR as these illustrate opportunities to improve health and reduce costs. Comprehensive data remain scarce; a recent World Health Organization (WHO) systematic review identified a “*lack of properly designed and conducted economic studies comparing the resource use associated with resistant versus non-resistant pathogens*” [1].

Studies to determine health outcomes of infections with community and hospital onset must adequately account for confounding, the timing of infection (time dependency) and simultaneous impact on risk of death and discharge (competing risks), but also analyse a sample of sufficient size to produce precise estimates [4,5]. Furthermore, although the major determinant of the economic burden of such infections from the hospital perspective is the number of bed-days they consume, it is challenging to produce an appropriate economic valuation of each marginal bed-day [6].

Given the widespread dissemination of meticillin resistance among *Staphylococcus aureus* and resistance to third-generation cephalosporins among Enterobacteriaceae [7], we focused on these bacteria and resistance phenotypes. We examined bloodstream infections (BSI) because of their relatively high incidence, clinical impact and diagnostic certainty. We were interested in costs from the hospital perspective because this is the perspective from which decisions must be made to allocate resources to interventions such as antimicrobial stewardship and infection control.

### Objectives

We sought to apply state-of-the-art methods to obtain unbiased and adjusted estimates of the excess length of stay (LOS), hospital mortality, and cost (from the hospital perspective) attributable to BSI caused by *S. aureus* and Enterobacteriaceae in European hospitals, and to compare the impact of antimicrobial non-susceptible versus susceptible strains.

## Methods

### Study design

We performed a multicentre, retrospective cohort study. The cohort consisted of all acute-care admissions at 10 European hospitals from 1 January 2010 to 31 December 2011. BSI were the time-varying exposure of interest and their impact on hospital mortality, LOS and cost was evaluated. Independent analyses were performed for BSI due to *S. aureus* and Enterobacteriaceae.

This report was formulated in accordance with the STROBE Statement [8].

### Setting

A convenience sample of 10 European hospitals participated: three from Italy, two each from Germany and the United Kingdom, and one each from France, Spain and Switzerland. These participants were selected from a list of interested sites using a questionnaire addressing microbiological methods and clinical informatics. Hospitals were eligible if able to extract the required data from institutional databases. All eligible hospitals were included.

### Participants

We retrospectively identified all inpatient acute-care episodes lasting more than one calendar day that started during the study period. We excluded ambulatory, hospital-in-the-home and non-acute care episodes as well as emergency consultations without consequent hospital admission. There was no age limit. For patients with multiple admissions during the study period, only the first admission was included.

### Exposures

We considered four exposures defined by causative bacteria and antimicrobial susceptibility. *Escherichia coli*, *Klebsiella* spp. or *Proteus* spp. strains causing BSI were classified as third-generation cephalosporin-susceptible Enterobacteriaceae (3GCSE) or third-generation cephalosporin-non-susceptible (3GCRE). Non-susceptibility to third-generation cephalosporins was defined as intermediate susceptibility or resistance to ceftazidime and/or one of cefotaxime, ceftriaxone or cefpodoxime. *S. aureus* strains causing BSI were classified as meticillin-susceptible (MSSA) or meticillin-resistant (MRSA). BSI was defined by one or more blood cultures with growth of the relevant bacteria.

### Outcomes

The two primary outcomes were hospital mortality and excess LOS in hospital. Excess LOS was used to estimate costs from the hospital perspective.

### Covariates

Baseline variables considered as potential confounders were age, sex, location prior to episode, elective/emergent admission, nights hospitalised in the previous 12 months in the same institution and 17 comorbidities [9]. The Charlson Comorbidity Index was computed for descriptive purposes, but comorbidities were included in the analyses as individual covariates. Two time-varying covariates were considered while patients were at risk for BSI: admission to an intensive care unit (ICU) and surgical procedure. To estimate the total impact of infection and avoid controlling for intermediates on the causal pathway, we did not adjust for events occurring after BSI onset, such as antibiotic exposure.

BSI were categorised as hospital-onset if detected after the first three inpatient calendar days [10], if the patient was transferred from a non-acute ward or another hospital, or if the patient was born during the current admission. All others were categorised as community-onset.

### Data collection

One investigator from each site was trained in standardised data collection. Information technicians from each participating hospital extracted data from the hospital databases. Comorbidities were extracted using a validated algorithm based on ICD-9-CM and ICD-10 codes [11]. Each dataset was reviewed for internal consistency and external plausibility by the central coordinating team, with potential errors triggering review by the local investigators.

### Microbiological methods

Antimicrobial susceptibility testing was performed as per routine laboratory methods at each hospital. All laboratories participated in national or international quality assurance programmes and adhered to contemporary guidelines from the following bodies: Clinical and Laboratory Standards Institute (CLSI) for seven sites), European Committee on Antimicrobial Susceptibility Testing (EUCAST) for three sites, Antibiogram Committee of the French Microbiology Society (CA-SFM) for one site, British Society for Antimicrobial Chemotherapy (BSAC) for one site, and Deutsche Industrie Norm (DIN)-Medizinische Mikrobiologie for one site. Three sites used more than one guideline during the study period. Nine sites performed one or more MRSA confirmatory tests: oxacillin minimum inhibitory concentration (MIC) test (n = 6), *mecA* PCR (n = 4), and penicillin binding protein 2a (PBP2a) agglutination (n = 4). The site that did not perform these tests used disc diffusion (BSAC protocol) and the VITEK2 system with the AST-P578 panel (bioMérieux, Lyon, France). Confirmatory testing for extended-spectrum beta-lactamase (ESBL) production was performed by seven sites but not included in our definition of third-generation cephalosporin susceptibility.

### Sample size

The sample size calculation was based on the estimated excess LOS for ESBL-positive BSI, informed by estimates from a pilot study [12]. We wished to find the number of infections such that, with a power of 80% and α equal to 5%, we could conclude that excess LOS was greater than excess LOS/2, an estimate of precision, i.e. to have sufficient power to detect a lower confidence limit of at least half of the point estimate. On the basis of incidence data from participating hospitals, we expected to include approximately 1,250 patients with BSI caused by 3GCRE, allowing estimates with good precision for an excess LOS of four days or more.

### Statistical analysis

#### Descriptive statistics

Continuous variables are summarised as median with 25%–75% percentile, ordinal variables as count with percentage. BSI incidence density was computed by dividing the number of events by the number of patient-days at risk.

#### Estimation of mortality and excess length of stay

**Figure 1:**
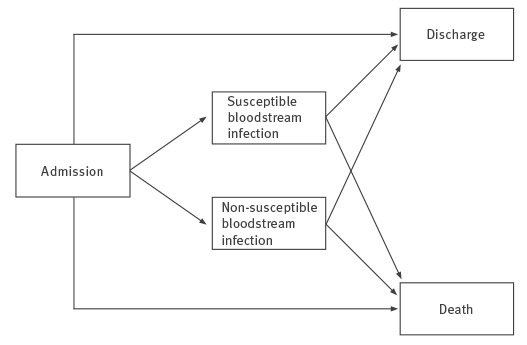
Multistate model adopted for the analysis of the burden of bloodstream infections caused by antimicrobial resistance, 2010–2011

Two important characteristics of this dataset were the inclusion of time-varying exposures (BSI, surgery and ICU admission) and competing risks (death and discharge alive). We adopted the multistate model illustrated in **Figure 1** to explicitly account for these characteristics [4]. Patients entered the initial state on admission to acute care and exited by entering one of two competing absorbing states (hospital death or discharge alive), with or without passing through one of two intermediate states (susceptible or non-susceptible BSI). Admissions were artificially right-censored at day 45 to reduce the influence of outliers. We reasoned that patients with such prolonged admissions were likely to remain hospitalised for other reasons not influenced by BSI.

Patients entered the initial state on admission to acute care unless the infection date was before or equal to the admission date, in which case the patient was assigned directly to the appropriate intermediate infected state. Patients exited by entering one of two competing absorbing states (death or discharge alive), with or without passing through one of two intermediate states (bloodstream infection caused by susceptible or non-susceptible pathogens). *Escherichia coli*, *Klebsiella* spp. or *Proteus* spp. were classified as susceptible or non-susceptible to third-generation cephalosporins. *Staphylococcus aureus* was classified as susceptible or non-susceptible to meticillin.

Cox proportional hazards models were used to compare the *daily* risk (hazard) of reaching the endpoint, i.e. hospital death, discharge alive, and the combined end-of-stay endpoint (hospital death or discharge alive), between the three groups of patients (non-susceptible BSI, susceptible BSI and uninfected subjects). We fitted three models for each possible pairwise comparison between these three groups. Model 1 only included infection status as time-dependent variable. Model 2 adjusted for age, sex, emergent/elective admission, nights hospitalised in the previous 12 months and comorbidities. Age was centred at the cohort mean and divided by 10. Each comorbidity was included as a separate indicator variable. Model 3 additionally adjusted for two time-dependent variables while patients were at risk for BSI: ICU admission and surgical procedure. All Cox models were stratified by hospital to account for the multicentre nature of this study by allowing for clustering effects and site-specific heterogeneity in baseline hazards. The proportional hazards assumption was checked by inspection of the Schoenfeld residuals. No major deviations were found.

Multistate models describe the *instantaneous* (in this case, daily) risk of transition between health states. The excess LOS associated with an infection was derived as a function of these transition probabilities [4]. We used the Aalen-Johansen estimators as a nonparametric estimator for the matrix of transition probabilities for all observed transition times [13]. The expected LOS (in days) was then computed by a function of the Aalen-Johansen estimator for the matrix of transition probabilities [4]. The expected change in LOS for each of the four BSI phenotypes was computed for each day of admission as the difference between the estimated LOS, given that BSI (the intermediate state) had or had not occurred up to that day. The overall change in LOS was computed as a weighted average of these quantities, with weighting determined by the observed distribution of time to BSI onset. The expected difference in LOS between susceptible and resistant infections was produced similarly, by computing for each day the difference between the estimated LOS, given that the susceptible or resistant BSI had occurred up to that day, then computing a weighted average of these quantities determined by the observed distribution of day of BSI onset. Standard errors and confidence intervals were derived by bootstrap re-sampling runs.

We adjusted excess LOS for the baseline covariates included in Model 2 using pseudo-observations [14]. Excess LOS was estimated for all possible subsets of the entire cohort created by removing a single patient. In each case, the excess LOS estimate was compared to the estimate derived from the full cohort; this difference or pseudo-observation contained information on the way in which patient-level covariates affected the LOS estimate. The pseudo-observations were then included in a generalised linear model with identity link and independent working covariance matrix to model the effect of covariates on the excess LOS. In practice, the regression coefficients were estimated using the generalized estimating equations approach with robust variance estimator to account for hospital-level clustering [15]. Time-dependent covariates (Model 3) were not included because this would have been difficult to implement and interpret. To reduce the influence of outliers, the original pseudo-observations were transformed using the cubic root function, similar to the common log transformation of LOS data but allowing for negative excess LOS.

#### Cost estimation

For each combination of bacterium and susceptibility, we computed the attributable cost of a single BSI from the hospital perspective as the product of excess LOS and the value of a bed-day [6]. We performed a Monte Carlo simulation with 10,000 samples to account for parameter uncertainty [16]. We used gamma probability distributions to represent the excess LOS associated with each BSI, fitting these distributions to the unadjusted estimate from the current study (Model 1) to best reflect our patient mix. We used log-normal distributions for two contrasting bed-day values, both obtained from the study hospitals as previously reported: an economic estimate of the opportunity cost of a bed-day obtained by contingent valuation and the accounting cost derived by dividing the total annual hospital budget by the number of bed-days supplied during the same period [17].

To estimate the annual hospital costs of each BSI, these marginal costs were multiplied by the expected number of BSI cases per year, as estimated for a hospital with 450,000 bed-days using incidence densities calculated in the analysis here below. Results are presented as median with 95% credible interval, to two significant figures. A full description of data sources and probability distributions can be obtained from the corresponding author.

The cost estimation was implemented in OpenBUGS, version 3.2.3. Other statistical analyses were performed using R, version 3.1.0 (R Foundation for Statistical Computing) including the etm, mvna, and survival packages [18].

### Ethics statement

This study was approved, with a waiver for individual informed consent, by the human research ethics committee at each institution. 

## Results

### Participants

Ten public hospitals provided a cohort of 867,977 acute-care episodes involving 606,649 patients (**Table 1**). Each patient’s first episode was included in the analysis.

**Table 1:** Characteristics of participating hospitals, analysis of the burden of bloodstream infections caused by antimicrobial resistance, 2010–2011 (n = 10)

Hospital	Country	Type	Acute beds	Study cohort	
				Admissions	Bed-days
1	France	Tertiary	1,200	64,071	424,361
2	Germany	Tertiary	1,200	67,094	456,564
3	Germany	Tertiary	1,332	59,517	431,384
4	Italy	Tertiary	1,100	89,401	677,788
5	Italy	Tertiary	1,050	53,947	373,665
6	Italy	Secondary	555	27,975	183,707
7	Switzerland	Tertiary	900	58,541	382,012
8	Spain	Tertiary	711	50,065	336,253
9	United Kingdom	Tertiary	1,050	92,569	423,534
10	United Kingdom	Tertiary	936	43,469	265,392
**Total**			**10,034**	**606,649**	**3,954,660**

Note: Admissions and bed-days relate to acute-care episodes from 2010 and 2011 included in the analysis. For patients with multiple admissions during the study period, only the first admission was included.

Median patient age at admission was 49 years (interquartile range (IQR): 28–69); 53% were female. Median LOS was four days (IQR: 2–7), and 588,118 (97%) patients were discharged alive. Of the remaining cohort, 10,419 (1.7%) died and 8,112 (1.3%) remained in hospital at the end of the study period (and underwent administrative censoring). Baseline characteristics are presented in **Table 2**.

**Table 2:** Characteristics of patients in *Staphylococcus aureus* and Enterobacteriaceae analyses, stratified by exposure to bloodstream infection in 10 European hospitals, 2010–2011 (n = 606,649^a^)

Characteristic	*Staphylococcus aureus* analysis						Enterobacteriaceae analysis					
	MRSA BSI		MSSA BSI		Non-infected		3GCRE BSI		3GCSE BSI		Non-infected	
	n = 163		n = 885		n = 604,797		n = 360		n = 2,100		n = 603,972	
	n	%	n	%	n	%	n	%	n	%	n	%
Demographics												
Male sex	98	60.1	529	59.8	286,857	47.4	206	57.2	1,051	50.0	286,492	47.4
Median age at enrolment (IQR)	71 (59–81)		63 (45–76)		49 (28–69)		70 (58–78)		70 (56–80)		49 (27–69)	
Hospitalisation in the previous 12 months												
Two or more admissions	15	9.2	40	4.5	15,708	2.6	23	6.4	112	5.3	15,660	2.6
Two or more nights hospitalised	24	14.7	72	8.1	37,468	6.2	46	12.8	229	10.9	37,354	6.2
Admission details												
Emergent admission	111	68.1	639	72.2	282,382	46.7	217	60.3	1,588	75.6	281,844	46.7
Provenance												
Home	134	82.2	734	82.9	500,693	82.8	303	84.2	1,848	88.0	499,942	82.8
Transfer from other hospital	16	9.8	73	8.2	16,930	2.8	21	5.8	75	3.6	16,927	2.8
Transfer from non-acute ward	2	1.2	11	1.2	2,722	0.5	1	0.3	26	1.2	2,713	0.4
Born this episode	3	1.8	7	0.8	47,414	7.8	3	0.8	29	1.4	47,397	7.8
Comorbidities												
Cardiovascular disease	8	4.9	50	5.6	15,192	2.5	10	2.8	97	4.6	15,160	2.5
Congestive heart failure	29	17.8	109	12.3	22,935	3.8	32	8.9	195	9.3	22,886	3.8
Peripheral vascular disease	17	10.4	65	7.3	16,515	2.7	9	2.5	89	4.2	16,509	2.7
Cerebrovascular disease	14	8.6	77	8.7	22,908	3.8	27	7.5	165	7.9	22,836	3.8
Dementia	9	5.5	14	1.6	5,759	1.0	7	1.9	64	3.0	5,743	1.0
COPD	7	4.3	40	4.5	25,701	4.2	16	4.4	112	5.3	25,647	4.2
Connective tissue disease	2	1.2	19	2.1	5,123	0.8	2	0.6	31	1.5	5,124	0.8
Peptic ulcer disease	1	0.6	15	1.7	2,527	0.4	7	1.9	27	1.3	2,513	0.4
Mild liver disease	9	5.5	76	8.6	12,541	2.1	31	8.6	150	7.1	12,478	2.1
Diabetes without end-organ damage	23	14.1	127	14.4	38,004	6.3	28	7.8	255	12.1	37,937	6.3
Diabetes with end-organ damage	7	4.3	37	4.2	7,901	1.3	7	1.9	60	2.9	7,886	1.3
Haemiplegia or paraplegia	6	3.7	44	5.0	7,374	1.2	12	3.3	55	2.6	7,363	1.2
Renal disease	26	16.0	110	12.4	21,309	3.5	30	8.3	233	11.1	21,231	3.5
Neoplasia	13	8.0	79	8.9	43,830	7.2	45	12.5	277	13.2	43,641	7.2
Metastatic cancer	2	1.2	35	4.0	16,393	2.7	24	6.7	119	5.7	16,302	2.7
Liver diseases	2	1.2	31	3.5	3,047	0.5	11	3.1	49	2.3	3,030	0.5
HIV	0	0.0	12	1.4	1,277	0.2	1	0.3	17	0.8	1,271	0.2
Age-adjusted Charlson comorbidity index, median (IQR)	4 (3–5)		3 (1–5)		1 (0–3)		4 (2–5)		4 (2–5)		1 (0–3)	
Primary diagnosis category												
Certain infectious and parasitic diseases	20	12.3	132	14.9	13,216	2.2	53	14.7	411	19.6	13,060	2.2
Neoplasms	10	6.1	61	6.9	56,345	9.3	41	11.4	225	10.7	56,165	9.3
Blood and blood-forming organs and certain disorders involving the immune mechanism	1	0.6	6	0.7	4,400	0.7	4	1.1	9	0.4	4,399	0.7
Endocrine, nutritional and metabolic diseases	7	4.3	16	1.8	14,320	2.4	4	1.1	23	1.1	14,320	2.4
Mental and behavioural disorders	1	0.6	5	0.6	6,270	1.0	7	1.9	17	0.8	6,255	1.0
Nervous system, eye and adnexa, ear and mastoid process	3	1.8	34	3.8	40,844	6.8	5	1.4	24	1.1	40,848	6.8
Circulatory system	35	21.5	201	22.7	80,361	13.3	53	14.7	221	10.5	80,324	13.3
Respiratory system	5	3.1	34	3.8	33,426	5.5	21	5.8	88	4.2	33,377	5.5
Digestive system	13	8.0	51	5.8	44,350	7.3	45	12.5	322	15.3	44,175	7.3
Skin and subcutaneous tissue	3	1.8	29	3.3	9,597	1.6	1	0.3	18	0.9	9,607	1.6
Musculoskeletal system and connective tissue	14	8.6	91	10.3	33,452	5.5	7	1.9	21	1.0	33,513	5.5
Genitourinary system	9	5.5	28	3.2	30,257	5.0	37	10.3	365	17.4	30,107	5.0
Pregnancy, childbirth and the puerperium	0	0.0	8	0.9	54,785	9.1	3	0.8	42	2.0	54,758	9.1
Certain conditions originating in the perinatal period	2	1.2	6	0.7	18,641	3.1	2	0.6	28	1.3	18,624	3.1
Congenital malformations, deformations and chromosomal abnormalities	2	1.2	7	0.8	12,357	2.0	1	0.3	16	0.8	12,350	2.0
Symptoms, signs and abnormal clinical and laboratory findings, not elsewhere classified	3	1.8	33	3.7	23,985	4.0	9	2.5	80	3.8	23,963	4.0
Injury, poisoning and certain other consequences of external causes	34	20.9	127	14.4	79,622	13.2	63	17.5	169	8.0	79,565	13.2
External causes of morbidity and mortality	0	0.0	3	0.3	85	0.0	0	0.0	1	0.0	87	0.0
Factors influencing health status and contact with health services	1	0.6	2	0.2	47,551	7.9	4	1.1	15	0.7	47,536	7.9
Epidemiological classification of BSI												
Hospital onset	101	62.0	434	49.0	NA		214	59.4	780	37.1	NA	
Community onset	62	38.0	451	51.0	NA		146	40.6	1,320	62.9	NA	
Interventions prior to BSI												
Surgical procedure	39	23.9	243	27.5	246,485	40.8	112	31.1	462	22.0	246,180	40.8
ICU admission	52	31.9	258	29.2	43,307	7.2	112	31.1	434	20.7	43,068	7.1

BSI: bloodstream infection; COPD: chronic obstructive pulmonary disease; HIV: human immunodeficiency virus; ICU: intensive care unit; IQR: interquartile range; MRSA/MSSA: meticillin-resistant/susceptible *Staphylococcus aureus*; NA: not applicable; 3GCRE/3GCSE: third-generation cephalosporin-resistant/susceptible Enterobacteriaceae.

^a^ Patients experiencing BSI caused by Enterobacteriaceae were censored from the S. aureus analysis on the day of the Enterobactericeae BSI. Patients experiencing BSI caused by Enterobacteriaceae on the day of admission were therefore excluded from the S. aureus analysis. The inverse applies for the Enterobacteriaceae analysis.

### BSI incidence

Of the 1,048 admissions during which *S. aureus* BSI were detected, 885 (84%) and 163 (16%) were due to MSSA and MRSA, respectively. The incidence density of *S. aureus* BSI was 0.269 episodes per 1,000 patient-days at risk: 0.227 and 0.042 episodes per 1,000 patient-days at risk for MSSA and MRSA BSI, respectively.

Of the 2,460 admissions during which Enterobacteriaceae BSI were detected, 2,100 (85%) and 360 (15%) were due to 3GCSE and 3GCRE, respectively. The incidence density of BSI due to Enterobacteriaceae was 0.631 episodes per 1,000 patient-days at risk: 0.538 and 0.092 episodes per 1,000 patient-days at risk for 3GCSE and 3GCRE BSI, respectively.

### Hospital mortality and discharge alive

In the *S. aureus* analysis, 149 (16.8%) and 36 (22.1%) patients with MSSA and MRSA BSI died in hospital, respectively, compared with 10,161 (1.7%) non-infected patients. In the Enterobacteriaceae analysis, 212 (10.1%) and 58 (16.1%) patients with 3GCSE and 3GCRE died in hospital, respectively, compared with 10,105 (1.7%) non-infected patients.

Results from the Cox proportional hazards analyses for death and discharge alive should be interpreted together (**Table 3**) [19].

**Table 3:** Results of proportional hazards models for hospital mortality and discharge alive, 10 European hospitals, 2010–2011 (n = 606,649)

			Mortality HR (95% CI)			Discharge alive HR (95% CI)		
Comparison	Exposure	Population	Model 1	Model 2	Model 3	Model 1	Model 2	Model 3
MSSA BSI vs non-infected	MSSA BSI	Hospitalised patients	2.58 (2.19–3.04)	2.41 (2.05–2.84)	1.81 (1.49–2.20)	0.34 (0.31–0.37)	0.38 (0.35–0.41)	0.54 (0.50–0.60)
MRSA BSI vs non-infected	MRSA BSI	Hospitalised patients	3.18 (2.29–4.42)	2.61 (1.88–3.63)	2.42 (1.66–3.51)	0.25 (0.20–0.32)	0.30 (0.24–0.38)	0.45 (0.36–0.58)
MRSA BSI vs MSSA BSI	Meticillin resistance	Patients with *S. aureus* BSI	1.19 (0.81–1.75)	1.20 (0.82–1.76)	1.26 (0.82–1.94)	0.74 (0.58–0.94)	0.73 (0.57, 0.94)	0.80 (0.61, 1.05)
3GCSE BSI vs non-infected	3GCSE BSI	Hospitalised patients	2.25 (1.96–2.58)	1.74 (1.51–1.99)	1.16 (0.98–1.36)	0.52 (0.49–0.54)	0.61 (0.58–0.64)	0.80 (0.75–0.84)
3GCRE BSI vs non-infected	3GCRE BSI	Hospitalised patients	2.88 (2.22–3.74)	2.25 (1.73–2.92)	1.80 (1.34–2.42)	0.37 (0.32–0.43)	0.43 (0.38–0.50)	0.57 (0.49–0.67)
3GCRE BSI vs 3GCSE BSI	3GC resistance	Patients with Enterobacteriaceae BSI	1.39 (1.02–1.90)	1.43 (1.05–1.96)	1.63 (1.13–2.35)	0.63 (0.55–0.73)	0.65 (0.56–0.75)	0.68 (0.57–0.81)

BSI: bloodstream infection; CI: confidence interval; HR: hazard ratio; MRSA/MSSA: meticillin-resistant/susceptible *Staphylococcus aureus*; 3GC: third-generation cephalosporins; 3GCRE/3GCSE: third-generation cephalosporin-resistant/susceptible Enterobacteriaceae.

Model 1: Susceptible and resistant BSI as time-dependent covariates (univariable analysis).

Model 2: As model 1 plus adjustment for age, sex, emergent/elective admission, nights hospitalised in the previous 12 months and comorbidities.

Model 3: As model 2 plus admission to intensive care and surgical procedures as time-dependent covariates.

When adjusted for potential confounders, all BSI except 3GCSE significantly increased the hazard of hospital death compared with non-infected patients. This effect was greater for BSI due to *S. aureus* than BSI due to Enterobacteriaceae. Moreover, all BSI strongly reduced the hazard of discharge alive after adjustment for confounders, meaning that patients with BSI stayed longer in hospital (discharge alive HR less than 1) and were exposed to an increased daily risk of death throughout this prolonged period (mortality HR greater than 1).

Among patients with BSI due to Enterobacteriaceae, third-generation cephalosporin resistance significantly increased the hazard of death compared with third-generation cephalosporin susceptibility (adjusted hazard ratio (aHR): 1.63; 95%CI: 1.13–2.35). In contrast, the trend for meticillin resistance to increase hazard of death amongst patients with *S. aureus* BSI did not reach statistical significance (aHR: 1.26; 95%CI: 0.82–1.94). Similarly, while third-generation cephalosporin resistance significantly decreased the hazard of discharge alive among patients with BSI due to Enterobacteriaceae, meticillin resistance showed only a trend in this direction among patients with BSI due to *S. aureus*.

### Excess length of stay

**Table 4** presents the impact of BSI on the combined end-of-stay endpoint (end-LOS HR) and excess LOS (in days) when compared with patients without BSI.

**Table 4:** Results of proportional hazards analysis for all-cause end-length of stay and excess length of stay estimates from multistate models, 10 European hospitals, 2010–2011 (n = 606,649)

			All-cause end-LOS HR (95% CI)			Excess LOS days (95% CI)	
Comparison	Exposure	Population	Model 1	Model 2	Model 3	Model 1	Model 2
MSSA BSI vs non-infected	MSSA BSI	Hospitalised patients	0.42 (0.39–0.45)	0.46 (0.43–0.49)	0.64 (0.59–0.69)	10.35 (9.44–11.26)	11.54 (11.45–11.63)
MRSA BSI vs non-infected	MRSA BSI	Hospitalised patients	0.36 (0.30–0.44)	0.42 (0.35–0.51)	0.61 (0.50–0.75)	12.22 (9.89–14.55)	13.33 (13.23–13.42)
MRSA BSI vs MSSA BSI	Meticillin resistance	Patients with *S. aureus* BSI	0.84 (0.68–1.03)	0.83 (0.67–1.02)	0.89 (0.71–1.12)	1.77 (-0.51–4.05)	2.54 (-3.19–8.27)
3GCSE BSI vs non-infected	3GCSE BSI	Hospitalised patients	0.57 (0.54–0.60)	0.66 (0.63–0.69)	0.84 (0.80–0.89)	4.36 (3.91–4.81)	5.87 (5.82–5.93)
3GCRE BSI vs non-infected	3GCRE BSI	Hospitalised patients	0.46 (0.41–0.52)	0.53 (0.47–0.60)	0.69 (0.60–0.79)	7.91 (6.66–9.16)	9.28 (9.20–9.35)
3GCRE BSI vs 3GCSE BSI	3GC resistance	Patients with Enterobacteriaceae BSI	0.72 (0.63–0.82)	0.73 (0.64–0.83)	0.78 (0.67–0.90)	3.53 (2.08–4.98)	4.89 (1.11–8.68)

BSI: bloodstream infection; CI: confidence interval; LOS: length of stay; MRSA/MSSA: meticillin-resistant/susceptible *Staphylococcus aureus*; 3GC: third-generation cephalosporins; 3GCRE/3GCSE: third-generation cephalosporin-resistant/susceptible Enterobacteriaceae.

Model 1: Susceptible and resistant BSI as time-dependent covariates (univariable analysis).

Model 2: As model 1 plus adjustment for age, sex, emergent/elective admission, nights hospitalised in the previous 12 months and comorbidities.

Model 3: As model 2 plus admission to intensive care and surgical procedures as time-dependent covariates.

**Figure 2:**
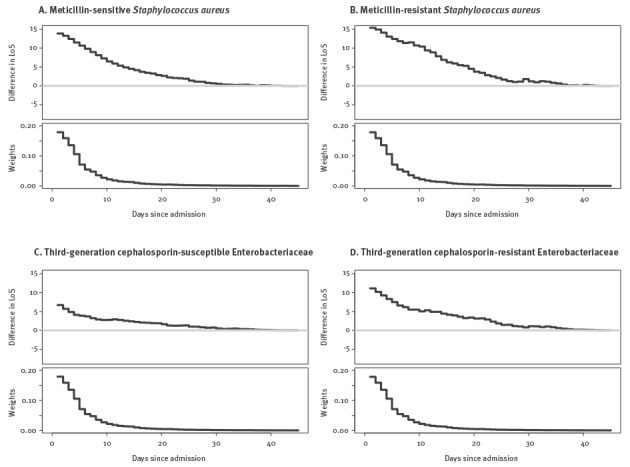
Results of multistate models to determine excess length of stay attributable to bloodstream infection caused by different combinations of bacteria and susceptibility, 10 European hospitals, 2010–2011 (n = 606,649)

All BSI reduced the daily all-cause hazard of discharge or death, i.e. led to prolonged hospital stay. This prolonging effect was greater for BSI due to *S. aureus* than for BSI due to Enterobacteriaceae, regardless of antimicrobial susceptibility status. For all types of BSI, diagnosis early during admission was associated with the greatest difference in LOS (**Figure 2**).

LOS: length of stay; MRSA/MSSA: meticillin-resistant/susceptible *Staphylococcus aureus*; 3GCRE/3GCSE: third-generation cephalosporin-resistant/susceptible Enterobacteriaceae.

The upper half in each panel (A-D) illustrates the relationship between the expected change in LOS associated with a BSI (computed daily by subtracting the LOS of patients that had not experienced BSI on that day from those who had) and timing of BSI onset (in days from admission). The lower half of each panel presents the weights used to compute the summary excess LOS, calculated using the observed relative frequency BSI onset each day. For all types of infections, early BSI was associated with the greatest difference in LOS.

**Table 4** also presents the end-LOS HR and excess LOS for BSI caused by resistant versus susceptible pathogens. While third-generation cephalosporin resistance significantly prolonged LOS amongst patients with BSI due to Enterobacteriaceae, meticillin resistance did not for the cohort of patients with *S. aureus* BSI.

The adjusted excess LOS estimate (Model 2) was taken from the model intercept, and should therefore be interpreted as the excess LOS caused by infection in a female patient with age equal to the mean age in the cohort, who has no comorbidities, has not been in hospital for the previous year, and was admitted electively. Increasing age, emergency admission, male sex, and all comorbidities except myocardial infarction decreased the excess length of stay associated with all four BSI types.

### Cost

The cost, from a hospital perspective, of each BSI and its annual cumulative incidence is presented in **Table 5**.

**Table 5:** Monte Carlo simulation results using economic and accounting bed-day values to estimate the cost of bloodstream infections, 10 European hospitals, 2010–2011 (n = 606,649)

Exposure	Population	Excess LOS per BSI days (95% CrI) ^a^	Expected annual cumulative incidence per hospital **^b^**	Estimated cost per infection EUR (95% CrI)		Estimated cost per hospital-year EUR 1,000 (95% CrI)	
				Economic costing ^c^	Accounting costing ^d^	Economic costing ^c^	Accounting costing ^d^
MSSA BSI	Hospitalised patients	10.3 (9.3–11.5)	102	760 (190–3,000)	9,500 (5,800–16,000)	77 (19–300)	970 (590–1,600)
MRSA BSI	Hospitalised patients	12.2 (9.9–14.7)	19	890 (220–3,600)	11,000 (6,600–19,000)	17 (4.1–67)	210 (130–360)
Meticillin resistance	Patients with *S. aureus* BSI	1.9 (−0.7 to 4.6)	NA	120 (−60 to 740)	1,600 (−700 to 5,000)	NA	NA
3GCSE BSI	Hospitalised patients	4.4 (3.9–4.9)	242	320 (80–1,300)	4,000 (2,400–6,700)	77 (19–300)	970 (590–1,600)
3GCRE BSI	Hospitalised patients	7.9 (6.6–9.4)	41	560 (140–2,300)	7,300 (4,300–12,000)	24 (5–94)	300 (180–510)
3GC resistance	Patients with Enterobacteriaceae BSI	3.5 (2.1–5.1)	NA	250 (60–1,100)	3,200 (1,600–6,000)	NA	NA

BSI: bloodstream infection; CrI: credible interval; LOS: length of stay; MRSA/MSSA: meticillin-resistant/susceptible *Staphylococcus aureus*; NA: not applicable; 3GC: third-generation cephalosporins; 3GCRE/3GCSE: third-generation cephalosporin-resistant/susceptible Enterobacteriaceae.

^a^ Output from probabilistic sensitivity analysis based on input distributions, reproduced to demonstrate consistency with estimates from the current study.

^b^ Estimated for a hospital with 450,000 bed-days annually (95% CrI not displayed because precision from the study cohort is such that no additional uncertainty is added to the model).

^c^ Employs the bed-day valuation derived from a contingent valuation survey that estimated the opportunity cost of each bed-day consumed by a patient with BSI.

^d^ Employs the bed-day valuation computed by dividing total hospital budget for one year by the number of bed-days supplied during the same period. Refer to [8] for further details. All costs are displayed at two significant figures.

While 3GCSE BSI was associated with the lowest per-infection cost (EUR 320; 95% credible interval (CrI): 80–1,300; or EUR 4,000; 95% CrI 2,400–6,700, using economic and accounting valuations, respectively), their relative frequency resulted in equal highest annual cost with MSSA (EUR 77,000; 95% CrI: 19,000–300,000; or EUR 970,000; 95% CrI: 590,000–1,600,000, using economic and accounting valuations, respectively).

## Discussion

Per infection, *S. aureus* BSI had a greater effect on mortality, LOS and cost than BSI due to Enterobacteriaceae. Meticillin resistance, however, did not significantly increase the hazard of death or further prolong the excess LOS amongst patients with *S. aureus* BSI. This contrasts with BSI due to Enterobacteriaceae, where third-generation-cephalosporin-resistance increased both the hazard of mortality and excess LOS compared with susceptible strains. Furthermore, the annual cost, from a hospital perspective, of BSI due Enterobacteriaceae was equivalent to the cost of *S. aureus* BSI because the higher incidence of the former balanced the greater per-infection impact of the latter.

This study incorporated several novel methodological approaches to the recently described challenges when estimating the impact of AMR [5]. Multistate modelling is an extension of survival analysis that permits explicit modelling of time-varying exposures and competing outcomes [4], but previous applications to hospital epidemiology have not addressed confounding. We employed the flexible pseudo-observation regression technique to adjust these estimates for time-invariant potential confounders [14]. We also formally computed the excess LOS due to infections caused by non-susceptible compared with susceptible pathogens [20] rather than heuristically extrapolating this as the difference between excess LOS associated with each infection type compared with non-infected controls. Inclusion of the entire cohort of acute inpatients from 10 hospitals over two years facilitated precise estimates and avoided selection bias at patient-level, a risk when using matched cohorts.

We used a previously reported economic valuation of the opportunity cost of hospital bed-days to translate excess LOS to cost of BSI from the hospital perspective [17], employing a Monte Carlo simulation to preserve uncertainty in this estimation. Substantially higher cost estimates were produced using an accounting bed-day value in order to demonstrate the importance of the costing approach used. Accounting values are readily obtained but only show what has historically been spent on a bed-day. As the majority of hospital costs are fixed, this figure does not represent an amount that could be recouped should the infection be avoided. We contend that economic values, based on the opportunity cost of occupied bed-days, are appropriate for making decisions from the hospital perspective about future resource allocation for infection control programmes [21]. The lower cost of BSI, and also of AMR, obtained using the economic valuation provides insight into the financial challenges faced by hospital leadership when considering such interventions under existing funding arrangements. While we used the unadjusted excess LOS for this estimation to best reflect the patient mix in our cohort, the adjusted results and covariate coefficients could be used to transfer our excess LOS estimate to settings with different patient mix.

A recent WHO systematic review of the published scientific literature on the health and economic impact of AMR concluded that the quality of evidence on hospital LOS and mortality was ‘very low’ in most cases [1]. It also identified a paucity of studies comparing hospital costs incurred by infection with resistant versus susceptible isolates of *E. coli* and *K. pneumoniae*. Our results are consistent with this review’s finding that third-generation cephalosporin resistance is associated with increased risk of mortality among patients infected with *E. coli* or *K. pneumoniae*. We found that third-generation cephalosporin resistance increased the hospital LOS associated with BSI caused by Enterobacteriaceae, while previous reports were ‘inconsistent’ for *K. pneumoniae* BSI and found no excess LOS for *E. coli* BSI*.* Our results do not support the review’s finding that infection with MRSA is associated with increased mortality and LOS compared with MSSA. Potential explanations include more appropriate empiric antibiotic therapy during our study compared with older studies and inflated estimation of excess LOS in previous studies due to time-dependent bias [5,6]. In addition, daily risk (or hazard) of death, as estimated here, can be expected to be smaller than the cumulative risks reported in the review. Although seemingly in contrast to older literature, our findings are consistent with another recent, large European multicentre study that found that meticillin resistance had no significant impact on mortality (adjusted hazard ratio (aHR), 1.1; 95% CI: 0.7–1.8) or excess LOS (0.6 days; 95% CI: −3.7 to 5.3), whereas third-generation cephalosporin resistance increased both risk of death (aHR: 2.9; 95% CI: 1.2–6.9) and excess LOS (5.0 days; 95% CI: 0.4–10.2) [22,23]. A similarly modest impact of AMR has been reported in the European ICU setting [24].

These data should be interpreted within the context of the study design. The dataset was extracted retrospectively from existing databases. Concerns regarding the quality of ICD coding data have been well described [25], although the Charlson comorbidity index derived from administrative databases has elsewhere proven superior to chart review [26]. We relied on routine antimicrobial susceptibility results performed by local laboratories using guidelines from five different organisations. However, for MRSA and 3GCRE, there should not be a major misclassification bias. We were unable to detect community-onset healthcare-acquired infection, however our primary results do not depend on this distinction. In addition, we could not include antibiotic exposure data. However, we consider delayed appropriate antimicrobial therapy to be on the causal pathway between antimicrobial resistance and the outcomes of interest [27], so exclusion of this information from our analysis is appropriate. We were unable to follow up patients post discharge, thus cannot report 30-day mortality or longer-term sepsis outcomes [28]. As with any observational study, we cannot exclude residual confounding. Our research question, however, is not amenable to an experimental study, and by accounting for time-dependent bias and important confounders, these results add to the existing literature. Finally, our study was designed to evaluate cost from the hospital perspective and addressed neither societal costs, macroeconomic indicators, nor the global health-economic implications of a post-antibiotic future [29,30].

This multicentre study, conducted in 10 European hospitals, could cautiously be extrapolated to large hospitals in other high-income settings, although the burden of BSI will clearly vary depending on incidence, treatment and hospital funding schemes. However, the current study did not address the lack of data in this field from low- and middle-income countries, where limited diagnostic and therapeutic resources, combined with lower proportion of gross domestic product available for healthcare, are likely to translate to a greater burden of disease.

Our data demonstrate the substantial health and economic burden imposed by BSI in European hospitals. Per infection, BSI caused by non-susceptible strains were associated with higher mortality risk and cost than susceptible strains. Given that BSI due to non-susceptible *S. aureus* and Enterobacteriaceae strains are likely to add to rather than replace those due to susceptible strains [31,32], the additional impact of AMR is substantial. However, the higher incidence of BSI due to susceptible strains means that these currently represent a greater health and economic burden than non-susceptible strains, emphasising the importance of surveillance and infection control policies that target *infections* rather than *resistance*.

## Notes

**TIMBER Study group members:** In addition to the named authors of this work, the TIMBER study group includes Cristina Masuet-Aumatell, PhD, and Marta Banqué Navarro, PhD (Hospital Universitari de Bellvitge, L’Hospitalet de Llobregat, Spain), and Chiara Falcone, MS (Papa Giovanni XXIII Hospital, Bergamo, Italy).
